# Lithium-Induced Chronic Kidney Disease in a Pediatric Patient

**DOI:** 10.1155/2019/5406482

**Published:** 2019-06-03

**Authors:** Neena Gupta, Meghan Gibson, Ellen C. Wallace

**Affiliations:** ^1^University of Massachusetts Memorial Children's Medical Center, University Campus, Department of Pediatrics, 55 Lake Avenue North, A2 210, Worcester, MA 01655, USA; ^2^Hasbro Children's Hospital, Department of Pediatrics, 593 Eddy Street, Providence, RI 02903, USA; ^3^University of Massachusetts Memorial Children's Medical Center, University Campus, Department of Radiology, 55 Lake Avenue North, Worcester, MA 01655, USA

## Abstract

Lithium-induced nephropathy usually manifests in adulthood as it develops slowly after many years of cumulative exposure. There is very limited information available in pediatric patients. Renal function monitoring and timely intervention is the key in preventing lithium-induced chronic kidney disease in these patients. We report a case of a 14-year-old boy who was on lithium for almost 9 years for his complex psychiatric illness. He presented with increased urinary frequency and nocturia. His serum creatinine increased to 1.15 mg/dL (estimated glomerular filtration rate or eGFR 53 ml/min/1.73 m^2^) from a baseline of 0.78 mg/dL (eGFR 86 ml/min/1.73 m^2^) a year prior to this presentation. Results of the imaging study were consistent with lithium-induced nephropathy. He was managed conservatively. His serum creatinine returned to baseline of 0.78 mg/dL after a year of discontinuation of lithium, consistent with mild chronic kidney disease. This case highlights the fact that lithium-induced chronic kidney disease can present in pediatric age group when lithium is initiated at a young age in children and that timely intervention may prevent further progression of renal damage. In addition to drug levels, routine monitoring of renal function during lithium therapy is essential.

## 1. Introduction

Lithium is a mood stabilizing agent which is used for the treatment of bipolar disorder. Clinical manifestations of lithium nephrotoxicity include acute kidney injury, chronic kidney disease (CKD), impaired urinary concentrating ability (nephrogenic diabetes insipidus), renal tubular acidosis, and nephrotic syndrome. CKD is commonly due to chronic tubulointerstitial nephropathy (CTIN) [1, 2]. The rate of progression of CKD is a function of the duration and cumulative dose of lithium therapy [3]. CKD is usually diagnosed in adulthood as it generally manifests after 10–20 years of lithium exposure [1, 2]. However, it is not uncommon to see children on lithium therapy. This can result in exposures of many years and renal injury before adulthood. Therefore, increased awareness amongst pediatric providers is necessary for timely diagnosis of renal injury. We report a case of CKD due to chronic tubulointerstitial nephropathy in a 14-year-old boy after 9 years of lithium treatment.

## 2. Case Report

**Figure 1:**
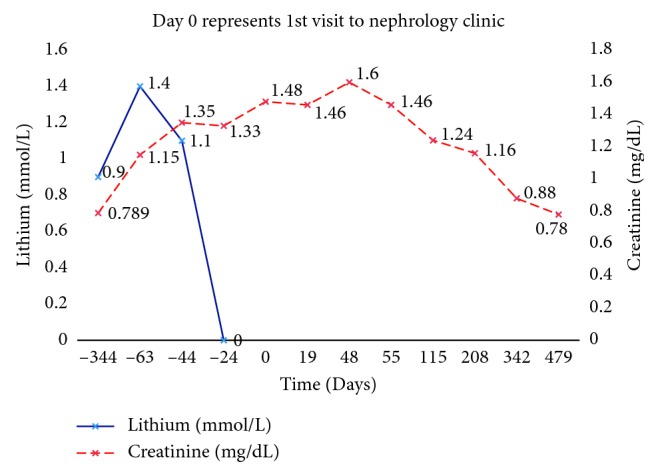
Time course of serum creatinine and lithium level.

**Figure 2:**
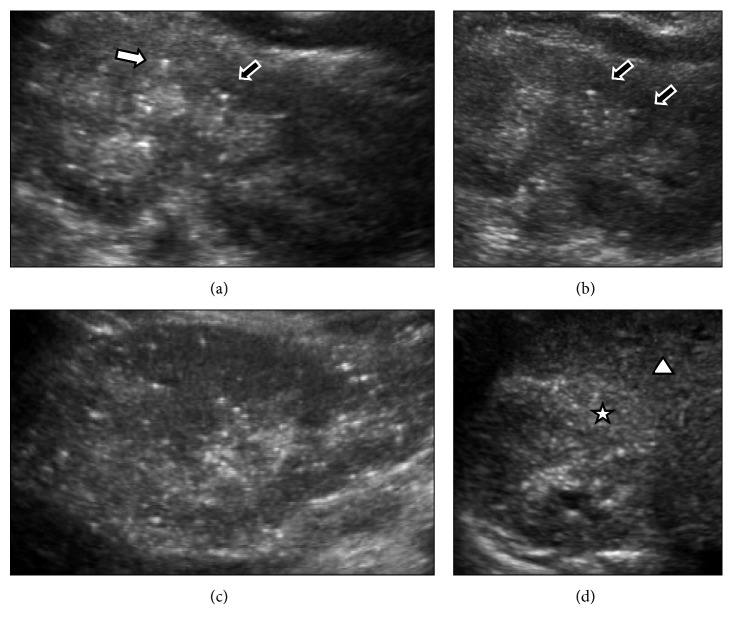
Renal ultrasound. (a) Sagittal left kidney: echogenic pyramids, punctuate hyperechoic foci in cortex, comet tail artifact (black arrow outlined in white), and small cyst with through transmission (white arrow with black outline). (b) Sagittal left kidney: echogenic pyramids with many punctuate hyperechogenicities. Two tiny sonolucent cysts with through transmission (black arrows with white outlines). (c) Sagittal right kidney: many punctuate cortical hyperechogenicities. (d) Transverse right kidney: generally increased cortical echogenicity (star) relative to adjacent liver (triangle).

A 14-year-old male with multiple psychiatric conditions, including bipolar disorder, attention deficit hyperactivity disorder, oppositional defiant disorder, posttraumatic stress disorder, and poor impulse control, presented to the primary care physician (PCP) with urinary frequency and nocturia of a month's duration. His medications included clonidine 0.1 mg three times daily, guanfacine 4 mg daily, bupropion sustained-release 150 mg daily, quetiapine 200 mg twice daily, and lithium 450 mg three times daily. Lithium was started when he was 5 years old. A year prior to his presentation, the lithium level was in the range of 0.9–1.1 mmol/L and serum creatinine was 0.78 mg/dL (estimated glomerular filtration rate or eGFR 86 ml/min/1.73 m^2^ based on the bedside Schwartz formula) [4]. Physical examination was unremarkable. Urinalysis revealed a specific gravity of 1.006, a pH of 6.0, but no hematuria or proteinuria. Blood tests showed a lithium level of 1.4 mmol/L, a TSH of 5.09 uIU/mL (normal 0.55–4.78 uIU/mL), a calcium of 11.1 mg/dL (normal 8.6–10.6 mg/dL), and a creatinine of 1.15 mg/dL. The PCP reduced the lithium dose to 450 mg twice daily and initiated levothyroxine for hypothyroidism. One week later, the lithium level reduced to 1.1 mmol/L. Over the next couple of weeks, the creatinine increased to 1.3 mg/dL. The PCP discontinued the lithium by tapering the dose over the next 3 weeks. Despite a very low lithium level of <0.1 mmol/L four weeks after discontinuation, the serum creatinine continued to increase, prompting a nephrology referral. At presentation to the nephrology clinic, physical examination was unremarkable. The creatinine was 1.46 mg/dL (Figure 1), calcium 13 mg/dL, ionized calcium 7.1 mg/dL (normal 4.6 to 5.3 mg/dL), phosphorus 3.3 mg/dL, PTH 3 pg/mL (normal 9–52 pg/mL), 25 hydroxy vitamin D 18 ng/mL (normal 30–100 ng/mL), alkaline phosphatase 110 IU/L, sodium 135 meq/L, potassium 4.8 meq/L, chloride 103 meq/L, bicarbonate 24 meq/L, BUN 20 mg/dL, albumin 4.5 g/dL, and TSH 0.42 uIU/mL. Urinalysis showed a specific gravity of 1.008, a pH of 6.5, but no hematuria or proteinuria. Spot urine protein to creatinine ratio was 183 mg/g Cr (normal <200 mg/g Cr), and calcium to creatinine ratio was 530 mg/g Cr (normal <200 mg/g Cr). A renal ultrasound (Figure 2) showed bilateral punctate hyperechogenic foci, small cysts, and extensive hyperechogenicities in both cortex and medulla.

The patient was managed conservatively with hydration and avoidance of nephrotoxic agents. His serum creatinine peaked at 1.6 mg/dL (eGFR 38 ml/min/1.73 m^2^) followed by a gradual return to baseline of 0.78 mg/dL (eGFR 86 ml/min/1.73 m^2^) at one year. Hypercalcemia and hypercalciuria also resolved with a serum calcium of 9.6 mg/dL, a PTH of 32 pg/mL, and a urine calcium to creatinine ratio of 55 mg/g Cr. Polyuria and nocturia persisted.

## 3. Discussion

Several studies have established the long-term risk of CKD in adult patients on lithium [2, 3, 5]. There are only a few case reports of lithium-induced nephropathy in children [6–8]. We report a 14-year-old patient who developed CKD, with a clinical and radiological picture suggesting CTIN, after a cumulative exposure of 9 years. Another unusual finding was hypercalcemia with suppressed PTH rather than hyperparathyroidism.

Clinical manifestations of lithium nephrotoxicity could be due to either chronic tubulointerstitial or glomerular injury or both. The most common finding on renal biopsy is CTIN in the form of interstitial fibrosis, dilated tubules, and microcysts. The clinical manifestations of lithium-induced CKD develop after 10–20 years of treatment; however, subclinical interstitial fibrosis can be seen on biopsy as early as 2 years [2]. Clinical presentations could include polyuria from impaired urinary concentrating ability (nephrogenic diabetes insipidus), low grade proteinuria, hematuria, and increased creatinine. In contrast, glomerular injury is less prominent, and individuals with glomerular toxicity usually present with nephrotic range proteinuria.

Several pathophysiologic mechanisms of lithium-induced nephropathy have been proposed. Lithium substitutes for sodium at epithelial sodium channels (ENaC) on the apical membrane of the collecting duct and gets transported into tubular cells and remains trapped, as it is a poor substrate for the sodium-potassium-ATPase pump on the basolateral membrane, leading to cytotoxic lithium concentrations [1]. Lithium inhibits adenylyl cyclase, thereby decreasing the expression of aquaporin 2 (AQP2) receptor for water transport. Lithium also inhibits glycogen synthase kinase type 3*β* (GSK-3*β*), an enzyme that controls the transport of water via the AQP2 channel. This results in a decreased response to antidiuretic hormone. Lithium also inhibits inositol monophosphate activity, leading to inositol depletion and cell cycle arrest [1].

The few pediatric case reports of lithium-induced nephropathy have shown nephrotic syndrome (glomerular injury) due to minimal change disease, focal segmental glomerulosclerosis, and membranous nephropathy [6–8]. One case report described a combination of glomerular and interstitial injury after use of lithium for 5.5 years [7]. Initial presentation in our patient suggested CKD due to CTIN, nephrogenic diabetes insipidus, and an element of acute kidney injury. The acute component was attributed to volume depletion and a lithium level higher than the therapeutic range. Lithium has a narrow therapeutic index with a commonly acceptable range of 0.6–1.0 mmol/L depending on severity of symptoms and age [9].

Our patient's low baseline eGFR prior to and after resolution of the acute component confirmed concurrent CKD. Renal ultrasound showed echogenic punctate foci and additional findings of microcysts with widespread involvement of the cortex and medulla. This pattern is most consistent with lithium-induced CKD. These punctate foci are thought to represent smaller microcysts [10] that are not detected by CT or MRI. The main differential diagnosis was nephrocalcinosis which usually causes echogenic and punctate foci predominantly in the medullary region.

Studies in adults show that the rate of progression of lithium-induced CKD correlates with the duration of treatment [3], but there is no literature available in pediatric patients. There is a mean annual decline in creatinine clearance of about 2.29 ml/min/year^3^ to 5 ml/min/year [5] in adult patients on chronic lithium therapy. In a review of 4879 adults without preexisting renal conditions, on lithium therapy for more than 10 years, an increase in median serum creatinine levels was noted within the first year. Also, 32% of the patients had eGFR below 60 ml/min per 1.73 m^2^ after 10 years or more on lithium and 5% had CKD stage 4-5 (eGFR <30 ml/min per 1.73 m^2^) [11].

Lithium-induced CKD patients may have some improvement in eGFR after stopping lithium [3], but there may be a “point of no return” whereby renal fibrosis is progressive despite stopping lithium [1–3]. In adults, it has been shown that a serum creatinine >2.5 mg/dL at the time of renal biopsy predicts progression to ESRD and drug interruption may only be beneficial for those with an estimated creatinine clearance >40 ml/min [1–3]. In our patient, the eGFR returned to a low baseline level after stopping lithium, indicating recovery from the acute lithium toxicity but persistent CKD.

Lithium is the most effective agent for bipolar disorder and may have to be continued in some patients if interruption of lithium results in detrimental psychiatric consequences. A close collaboration among PCP, psychiatrists, and nephrologists should help in balancing the risk of suicide, psychiatric relapses, and progressive renal damage, especially in patients who have already developed CKD [12].

**Figure 3:**
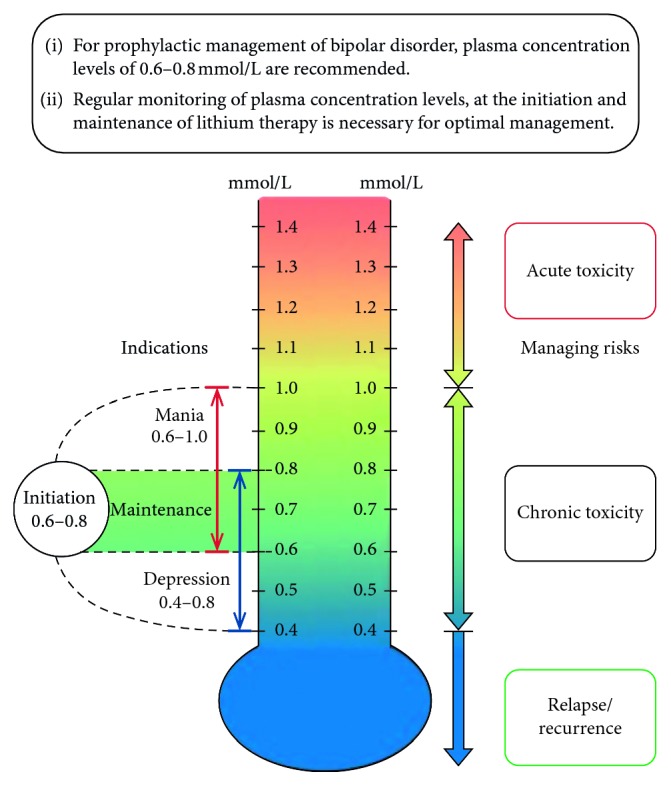
Lithiumeter (adapted from Malhi G. S. et al, Journal of Affective Disorders, 2017; 217–266).

All of the professional guidelines agree on the need to monitor kidney function periodically, although the recommendations vary [9]. The American Psychiatric Association recommends serum creatinine measurement every 2–3 months during the first 6 months of treatment and then every 6–12 months [13]. Chronic lithium toxicity can develop over time despite lithium levels in the recommended therapeutic range (Figure 3) [9].

Our patient also had hypercalcemia with suppressed PTH. We excluded common causes based on lack of exogenous intake and lab findings. Hypercalcemia resolved after stopping lithium. Lithium-induced hypercalcemia has usually been attributed to hyperparathyroidism. However, in published studies, the prevalence of hypercalcemia is much higher than the prevalence of hyperparathyroidism in such patients which suggests that mechanisms other than hyperparathyroidism may be involved [14].

To summarize, this case report shows that lithium-induced CKD can develop in a pediatric patient. Providers should vigilantly monitor renal function in children on lithium therapy.

## Abbreviation

eGFR: Estimated glomerular filtration rate
CKD: Chronic kidney disease
CTIN: Chronic tubulointerstitial nephropathy
PCP: Primary care physician
PTH: Parathyroid hormone
TSH: Thyroid-stimulating hormone
ENaC: Epithelial sodium channels
AQP2: Aquaporin 2
GSK-3*β*: Glycogen synthase kinase type 3*β*.
